# Development of Thickness-Dependent Predictive Methods for the Estimation of the CIEL*a*b* Color Coordinates of Monolithic and Layered Dental Resin Composites

**DOI:** 10.3390/ma16020761

**Published:** 2023-01-12

**Authors:** Maria Tejada-Casado, Razvan Ghinea, María M. Pérez, Javier Ruiz-López, Henning Lübbe, Luis Javier Herrera

**Affiliations:** 1 Department of Optics, Faculty of Science, Campus Fuentenueva, Edificio Mecenas, s/n., University of Granada, ibsGranada, 18071 Granada, Spain; 2 Instituto de Investigación Biosanitaria ibs.GRANADA, 18011 Granada, Spain; 3 Department of Physics, Faculty of Sciences, University of Craiova, 13 AI Cuza Street, 200585 Craiova, Romania; 4 Vita Zahnfabrik H. Rauter GmbH & Co. KG, Ballyweg 6, 79713 Bad-Säckingen, Germany; 5 Computer Architecture and Technology Department, University of Granada, 18071 Granada, Spain

**Keywords:** color prediction, linear regression, color thresholds, dental resin composites

## Abstract

Usually, dentin and enamel shades are layered in dental restorations with the purpose of mimicking the natural appearance of teeth. The main objective of this study was to develop and assess accuracy of a color-prediction method for both monolithic and layered dental resin-based composites with varying shades and under different illuminants. A total of 15 different shades of VITAPAN Excell, VITAPAN Dentine and VITA Physiodens as well as VITA Enamel of five different thicknesses (0.5–2.5 mm range) were used to manufacture monolithic and layered samples. A non-contact spectroradiometer with CIE $45^{\circ }/0^{\circ }$ geometry was used to measure the color of all samples over a standard ceramic black background. Second-degree polynomial regression was used as predictive method for CIE-L*a*b* color coordinates. Performance of predictive models was tested using the CIEDE2000 total color difference formula ($\Delta E_{00}$), while accuracy was evaluated by comparative assessment of $\Delta E_{00}$ with corresponding 50:50% acceptability ($AT_{00}$) and perceptibly ($PT_{00}$) thresholds for dentistry. A mean color difference between measured (real) and predicted color of $\Delta E_{00}=1.71$, with 62.86% of the color differences below $AT_{00}$ and 28.57% below $PT_{00}$, was registered for monolithic samples. For bi-layered samples, the mean color difference was roughly $\Delta E_{00}=0.50$, with generally 100% and more than 85% of the estimations below $AT_{00}$ and $PT_{00}$, respectively. The predictive method allowed highly accurate color estimations for both monolithic and layered dental resin-based composites with varying thicknesses and under different illuminations. These results could be useful to maximize the clinical success of dental restorations.

## 1. Introduction

The goal of any dental restoration is to create a natural appearance that is highly aesthetic while remaining functional [1]. Among all available current dental restorative materials, resin composites have been widely used for anterior restorations because of their exceptional aesthetics and durability [2,3,4,5]. Since natural teeth consist of different layers (i.e., enamel, dentine and pulp), dental restorations must also replicate this pattern in order to achieve an optimal result, and this is normally achieved through layering techniques of different materials [6]. Often, the natural appearance of a dental structure cannot be mimicked with a single shade; therefore, it is difficult to achieve an optimal aesthetic outcome. In this sense, in the case of direct restorations, to obtain a proper color match with the natural tooth structures that surround the cavity, the use of two different types of resin composites (one for replacing dentin and one for replacing enamel) was reported to provide satisfactory results [7]. Moreover, the thicknesses of the different layers within the natural teeth are not constant across the entire dental structure, and vary depending on the tooth area. Therefore, the final color of a direct dental restoration is the result of the interaction of several layers of different restorative materials with varying shades and thicknesses [8,9,10].

Finding the best dentine and enamel shades combination is not an easy task. In most clinical settings, visual comparisons with the tabs from available commercial dental shade guides is the main approach when performing a shade match between a dental restoration and natural tooth color. However, when performing this type of visual assessment, the variability among different shade guides, the background or the lighting conditions are all parameters that have the potential to influence the final shade selection [11,12,13].

In dentistry, the CIELAB color space (CIE—International Comission on Illumination) and its associated color coordinates—CIE-L*, CIE-a* and CIE-b*—are extensively used [14,15,16,17]. The values of these coordinates are strongly affected by the illuminant being used for their computation, and changes in lighting alter the perceived color of most materials, as has also been proved for dental materials [16]. For this reason, the reflectance spectrum of an object is known as the best way to describe its color. Nonetheless, devices that are able to properly measure reflectance spectrum are usually very expensive and thus they are mostly used for research purposes only [18,19,20,21,22,23]. In order to introduce objective color measurements to the dental field and make the shade-matching process more accurate and not so highly subjective, simpler measuring devices, such as spectrophotometers or colorimeters [24,25,26], have been introduced within the clinical practice. These commercial devices usually provide measurements of the CIE-L*a*b* color coordinates [25,27,28]. Therefore, if these parameters can be predicted with a certain accuracy, the trial-and-error in the shade-matching process and the aesthetic outcome of dental restorations will be considerably improved.

Color prediction is among the fastest growing research areas in dentistry [17]. In recent years, experimental dental resin-based composites (DRC) reflectance data was succesfully predicted using multiple nonlinear regression models [29]. Also, the Kubelka–Munk model has proven to be valid for color prediction of single [30,31] and layered DRC materials [30]. Furthermore, recent studies have used Principal Components Analysis (PCA)-based predictive algorithms for reflectance reconstruction and color estimation of monolithic [32] layered [33] DRC samples of different shades and varying thicknesses. However, the fundamentals of these studies are based on spectral measurements, that, as mentioned before, are barely available in clinical practice. Linear regression models have been widely used for color prediction in dentistry. Few studies [34,35] proposed color estimations of CIE-L*a*b* color coordinates of natural dental structures using data measured with clinical devices. These studies used both linear and non-linear regression methods, but the performance of the proposed predictive algorithms was prone to improvement. This technique has also been used to evaluate the role of enamel thickness and predict refractive index on human tooth color [36], while other authors implemented a Multiple Linear Regression (MLR) method to predict the CIE-L*a*b* values of dental ceramics with different pigment concentrations [37], and evaluated its performance versus the Kubelka–Munk theory approach [38]. To the best of our knowledge, there are no studies that aimed for predictions of CIE-L*a*b* values of dental materials.

Therefore, the main objective of this study is to develop and assess the accuracy of a prediction method for the CIE-L*a*b* chromaticity coordinates of both monolithic and layered dental resin-based composites (DRC) with varying shade and under different illuminants.

In the present study, the following research hypotheses were tested: (1) linear regression-based L*a*b* prediction algorithms can be used to estimate the CIE-L*a*b* color coordinates of monolithic and layered dental samples under different illuminants with precise results, (2) there are differences in estimation accuracy for monolithic and layered samples and (3) the performance of the proposed method will be affected by the use of different types or shades of DRC.

## 2. Materials and Methods

### 2.1. Specimen Preparation

Monolithic DRC samples of different materials, shades and thicknesses were analyzed in the present study (Table 1). Three different dentine types—VITAPAN Excell (VE), VITAPAN Dentine (VD) and VITA Physiodens (VP) (Vita Zahnfabrik, Bad Säckingen, Germany)—and one enamel—VITA Enamel (EN) (Vita Zahnfabrik, Bad Säckingen, Germany)—were prepared.

According to manufacturer’s specifications, the samples were prepared by polymerization of dental masses under combined heat-and-pressure treatment. Steel molds, with defined nests of 15 mm diameter, were filled with the different masses and subjected to a temperature of 180 °C for 3 mins under defined pressure, heating and cooling rates in a conventional transfer press. Then, the samples were ground on a JUNG surface grinder (United Grinding, Miamisburg, OH, USA) to specific clinically relevant thicknesses of 0.7 mm, 1.0 mm, 1.5 mm, 2.0 mm and 2.5 mm with a tolerance of ±0.1 mm [39]. All sample thicknesses were measured with a digital caliper (Mitutoyo, Europe GmbH, Mainz-Kastel, Germany) at three different specimen spots. Next, they were polished with a conventional grinding machine and 9 μm diamond paste by the same trained operator. Finally, they were cleaned from debris in an ultrasonic bath with distilled water (Elmasonic S30H, Elma Schmidbauer, Singen, Germany) for 10 min and dried with gauze and compressed air. For each shade and thickness one sample was fabricated.

The performance of the proposed method was tested for both monolithic and layered samples. For the single layers, shades A1, A2, A3, A3.5, B2, C2 and D2 of VD, were used. The layered samples were obtained by pressure bonding a dentine single layer with an enamel single layer. The highly accurate manufacturing process resulted in excellent quality samples with completely flat surfaces, which allowed for a perfect coupling between the two layers. To be able to evaluate the performance of the predictive method both as a function of DRC type and shade, samples of A2 shade of VD, VE and VP composite materials, as well as samples of different dentine shades—S1, S2, S3, S4 and S5—with different lightness, were fabricated. According to manufacturer recommendations, each dentine composite has to be associated with a specific enamel shade. In this sense, samples corresponding to three enamel (EN) shades were also manufactured—EN1, EN2 and EN3 (Table 1). This resulted in a total of 8 different DRC systems (dentine–enamel combination) used in this study, as follows:

- Testing according to DRC type: VE-A2+EN1, VD-A2+EN3, VP-A2+EN3;
- Testing according to DRC shade: VE-S1+EN1, VE-S2+EN1, VE-S3+EN2, VE-S4+EN2 and VE-S5+EN2, being S1 and S2 light shades; S3 and S4 intermediate shades and S5 a dark shade.

### 2.2. Color Measurements

The device used for measuring the spectral reflectance spectrum (380–780 nm) of both monolithic and layered samples consisted of a spectroradiometer (SpectraScan PR 670, Photo Research, Syracuse, NY, USA), a research grade light source (Fiber-coupled Xe-Arc; 66485-300, Newport Corporation, Irvine, CA, USA) and a spectrally calibrated reflectance standard (SRS-3, Photo Research, Syracuse, NY, USA). All samples were positioned against a standard ceramic black background (Ceramic color standards CCSII, Lucideon, Staffordshire, UK; L* = 23.3; a* = −0.3; b* = −1.0) and measured in a completely dark room to simulate the darkness in the oral cavity. The PR 670 was placed 40 cm away from the samples and a $45^{\circ }/0^{\circ }$ illuminating/measuring geometry, as recommended by the CIE [40], was used. For each specimen, three repeated reflectance measurements—without replacement—were performed and the results were averaged into a single mean value.

As mentioned before, color is strongly dependant on the illumination being used for its computation. In order to also test the performance of our method under different illuminants, the spectral reflectance measurements were converted into CIELAB color coordinates using the CIE $2^{\circ }$ Standard Observer and three different illuminants: CIE D65 Standard Illuminant, as the illuminant recommended by the CIE [40]; CIE D55 Standard Illuminant, because it is the most used among the clinical devices [41]; and LED-B1, because among the new LED standard illuminants proposed by the CIE it is the closest in terms of CCT and xy-chromaticity coordinates to D65 and D55 [40,42].

### 2.3. Computational Method

In order to develop and evaluate a predictive method, the samples were divided into training and testing groups. Computed CIE-L*a*b* coordinates that corresponded to samples included in the training set were used to build the regression models, while those that corresponded to samples included in the testing set were used exclusively for testing appropriate functioning and accuracy of the predictive model. The approach used for training and testing sets selection was different for monolithic and layered samples.

**Figure 1:**
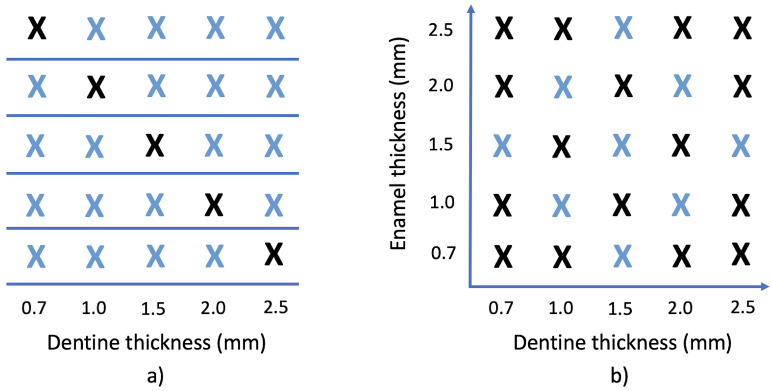
Samples included in the training set (blue) and testing set (black). (**a**) For monolithic samples, each thickness is used as a test sample while the other four thicknesses are included in the training set. (**b**) For bi-layered samples, 25 dentine + enamel combinations are obtained and divided into 9-samples training set and 16-samples testing set.

For single layer samples, given a total of 5 different samples (i.e., 5 different thicknesses) for each VP dentine shade, the samples were divided so that the CIE-L*a*b* coordinates of one of the dental samples were predicted using exclusively the samples corresponding to the same shade but with different thickness as training set. This separation into training and testing groups is repeated for all thicknesses of each shade, so that all samples are part of each set but never belong to both at the same time. A diagram of this division is shown in Figure 1.

In the case of the layered samples, a total of 25 enamel–dentin layered combinations for each DRC system were obtained (5 different samples of dentine layer ×5 different samples of enamel layer). The 25 layered specimens were thereafter distributed into training and testing sets. Other studies [33] have shown that, for a similar configuration, a training set of 9 samples is optimal to achieve very good color estimations. Therefore, in the present study, 9 samples heuristically selected were also included in the training set, while the other 16 were used for testing the predictive method. The training and testing sets arrangements are shown in Figure 1.

Linear regression models [43,44,45] were used to predict each CIE-L*, CIE-a* and CIE-b* coordinates of monolithic and layered samples of different thicknesses. Since the coordinates were predicted individually, three different models were computed for each test sample. Preliminary tests revealed that best performance was achieved when using 2nd degree polynomial to find the best-fit-curve and best-fit-surface for monolithic and layered samples, respectively.

For single layer samples, the equation describing the models is:

(1) $$f\left(x\right)=p_{1}x^{2}+p_{2}x+p_{3}$$

where, *f* is the predicted CIE-L*, CIE-a* or CIE-b* values, *x* corresponds to the sample thickness, and $p_{1}$ to $p_{3}$ are the parameters of the model.

For layered samples, the surface is defined as:

(2) $$s\left(x,y\right)=p_{1}x^{2}+p_{2}y^{2}+p_{3}xy+p_{4}x+p_{5}y+p_{6}$$

where, *s* is the predicted CIE-L*, CIE-a* or CIE-b* coordinate value, *x* and *y* correspond to the dentine and enamel thicknesses respectively and $p_{1}$ to $p_{6}$ are the parameters of the models.

**Figure 2:**
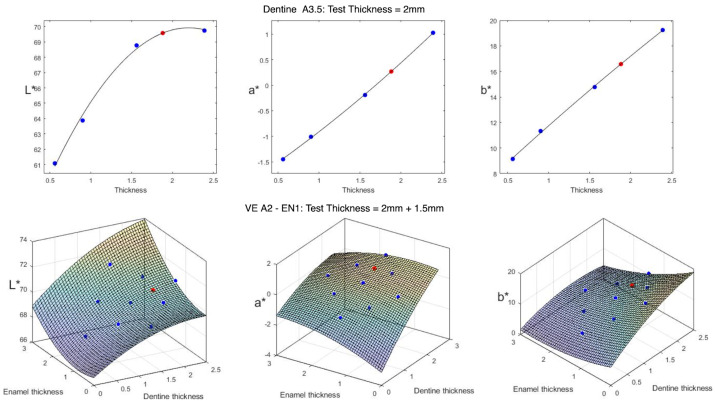
Examples of best-fit-curve (**top**) and best-fit-surface (**bottom**) for the CIE-L*, CIE-a* and CIE-b* color coordinates for monolithic and layered samples. Blue dots represent the values corresponding to the training set samples and red dots the values predicted by the model for a specific thickness.

In order to solve both problems, specific functions were implemented in MATLAB (MathWorks, Natick, MA, USA) to compute the linear least squares for monolithic and layered test samples, respectively. Once these curves and surfaces are determined, the CIE-L*, CIE-a* or CIE-b* values corresponding to any given thickness can be easily extracted. An example of this procedure is shown in Figure 2.

### 2.4. Color Difference Evaluation

Total color differences between measured and predicted values for each sample in the testing group were computed using the CIEDE2000 ($\Delta E_{00}$) [40] total color difference formula [46], as shown in Equation (3):

(3) $$\Delta E_{00}={\left[{\left(\frac{\Delta L^{\prime }}{K_{L}S_{L}}\right)}^{2}+{\left(\frac{\Delta C^{\prime }}{K_{C}S_{C}}\right)}^{2}+{\left(\frac{\Delta H^{\prime }}{K_{H}S_{H}}\right)}^{2}+R_{T}\left(\frac{\Delta C^{\prime }}{K_{C}S_{C}}\right)\left(\frac{\Delta H^{\prime }}{K_{H}S_{H}}\right)\right]}^{\frac{1}{2}}$$

$\Delta E_{00}$ values were comparatively evaluated with their corresponding 50:50% perceptibility ($PT_{00}$) and acceptability ($AT_{00}$) color difference thresholds for dentistry, as recommended by the ISO/TR 28642:2016 [47]. For dentistry, these thresholds were established as 50:50% $PT_{00}=0.8$ and 50:50% $AT_{00}=1.8$ [48,49], and have been largely used in dental research as a standard for color evaluation [50,51,52,53,54].

## 3. Results

In this study, linear regression models were used to predict the CIE-L*, CIE-a* and CIE-b* color coordinates for different sets of monolithic and layered samples of different thicknesses, shades and materials.

As previously described, the CIE-L*a*b* chromaticity coordinates were computed under different illuminants (D65, D55 and LED-B5). According to our results, the performance of the predictive methods was almost identical for all three illuminations tested, with total mean color differences between measured and predicted CIE-L*a*b* values for the whole data set (including monolithic and layered) of $\Delta E_{00}=0.74$, $\Delta E_{00}=0.75$ and $\Delta E_{00}=0.74$, for D65, D55 and LED-B5, respectively. When analyzing the percentage of color estimations that returned a color difference below the acceptability threshold of $AT_{00}\left(\%\right)$, it was found that, for the three illuminants tested, in 91.4% of the cases, color differences between real and predicted values were lower than $AT_{00}$. In the case of $PT_{00}\left(\%\right)$ the percentage of color differences between real and predicted values that are imperceptible to an average observer varied slightly between the different illuminants tested, being 72.4%, 73.0% and 71.8%, for D65, D55 and LED-B5, respectively. In this regard, for presentation purposes and simplicity, only the results corresponding to the CIE-L*a*b* values computed for the Standard D65 Illuminant are further presented for a deeper analysis, as it is also the recommended illuminant by the CIE [40].

Table 2 and Table 3 show the $\Delta E_{00}$ color differences between measured and predicted CIE-L*a*b* values for monolithic and layered samples, respectively. For the single layers, the values presented in Table 2 correspond to those of a certain test thickness when the other four samples of the same shade are being used to build the model. For the layered samples, the presented values (Table 3) correspond to the samples included in the testing set, as previously described in Section 2.3.

When dealing with predicted data, different modelling behaviours can be expected for the estimation of the CIE-L*a*b* values of a test sample whose thickness falls outside (extrapolation) or within the range of available training samples (interpolation). Thus, in order to assess the prediction capability of our method when out-of-the-range predictions are avoided, two different methods of analysis have been performed for the monolithic samples. For the extrapolation approach, all samples presented in the testing set are used in the evaluation; while for the interpolation approach, the analysis is limited only to those samples that fall within the cloud of available data (samples of 2.0 mm, 1.5 mm and 1.0 mm). The means and standard deviations of the performance metrics (ΔL*, Δa*, Δb* and $\Delta E_{00}$), as well as the percentage of samples lower than $AT_{00}$ and $PT_{00}$ for all shades and thicknesses, are presented in Table 4, for both extrapolation and interpolation approaches.

For layered samples, the performance of the method has been assessed both as a function of DRC type and shade lightness. As for the monolithic samples, the measured and predicted CIE-L*a*b* values were compared using different color metrics (ΔL*, Δa*, Δb* and $\Delta E_{00}$). The means and standard deviations, as well as the percentage of samples with color differences between measured and predicted values lower than $AT_{00}$ and $PT_{00}$, are presented in Table 5 and Table 6, respectively.

## 4. Discussion

In this study, linear regression predictive models were proposed for color estimation of monolithic and layered samples of different DRC materials and shades. The CIE-L*a*b* chromaticity coordinates of monolithic samples of five different thicknesses ranging from 0.7–2.5 mm corresponding to the different materials and shades used in this study were computed according to the CIE $2^{\circ }$ Standard Observer and three different illuminants [40]. Color differences between real measured values and estimated values were calculated using the CIEDE2000 color difference formula, as it is already largely implemented in dental color research [16,18,19,20,21,22,23] and it has been proven to more accurately fit human visual perception [46]. The results have been evaluated according to the 50:50% perceptibility and acceptability thresholds established for dentistry and dental applications ($PT_{00}=0.8$ and $AT_{00}=1.8$) [48,49].

Some studies have estimated CIE-L*a*b* values of dental natural structures based on data obtained with clinical color measurements devices [34,35]. However, their predictive models focused on color of natural teeth with respect to age and gender but not on dental materials, which is the main interest in dental restorations. Many existing studies confirmed that the color of different dental materials can be estimated using different predictive approaches [29,30,31,32,33]. Notably, all the proposed predictive models are based on acquired spectral data, which is not provided by most of the clinically available color measurement devices [24,25,26]. In this sense, an advantage of the predictive method proposed in this study is that it is based exclusively on CIE-L*a*b* coordinates, making it easier to implement and deploy in a real clinical scenario. Additionally, a wide variety of shades (including light, intermediate and dark colors) and clinically relevant thicknesses were used in the present study, which enabled a more comprehensive analysis of the performance of the proposed predictive algorithms with respect to different parameters.

Based on the results of this study, when both monolithic and layered samples corresponding to all shades and thicknesses were considered, the total mean color difference between the predicted and measured (real) data was roughly $\Delta E_{00}=0.74$, with 91.4% of the estimations exhibiting color differences lower than $AT_{00}$ (and approximately 72% lower than $PT_{00}$).

In a recent study [16], it was stated that color gamuts of a set of teeth, tested under several illuminants (including D65 and LED-B5, among others), exhibited similar shape and volume in CIELAB color space, but their centers of gravity shifted in different directions. Considering that the sensitivity of the human visual system also changes with illumination [55] and that the CIEDE2000 has been designed to fit more accurately with visual perception [46], a change of illumination could lead to higher $\Delta E_{00}$ between measured and estimated CIE-L*a*b* values. However, in the present study, very similar results were obtained independently of the illuminant considered (CIE D65, D55 ord LED-B5), which proves the robustness of the proposed predictive method and ensures satisfactory outcomes for its implementation in different settings. Therefore, the first research hypothesis was accepted since precise color coordinates estimations can be achieved for monolithic and layered samples using the proposed method, as shown by the low $\Delta E_{00}$ values, as exhibited in Table 2 and Table 3.

When comparing estimations for monolithic and layered samples, it was found that slightly better values were obtained for the later ones. At first sight, this might seem surprising, as predicting color data of monolithic samples should be an easier task and therefore lead to better results (in terms of predictive accuracy), since these samples are easier to fabricate and the predictive method is designed for two-dimensional data versus the three dimensions needed for the layered samples. However, the differences in performance between the monolithic and layered samples might be explained by the size of the training sets. In a study [33] where reflectance data and color of a set of DRC materials was estimated with predictive algorithms, it was proved that the sample size of the training set strongly affected the performance of the proposed predictive models. In this regard, in the present study, the training set consisted of only four samples for each monolithic shade group, while for the layered specimens the training set was composed of nine samples (Figure 1). In terms of color differences between predicted and real measured data, in the present study, 62.86% and 28.57% of the estimations showed color differences lower than $AT_{00}$ and $PT_{00}$, respectively, for training sets of four samples, as the ones used for the monolithic samples (Table 4). These results are in accordance with previous studies that predicted reflectance data and color of a set of DRC materials for training sets of five samples [33], where they reported between 75–100% of the color differences between predicted and real measured data below $AT_{00}$, and 5–75% below $PT_{00}$.

When a predicted data point falls outside the cloud defined by the set of data (training) points (i.e., extrapolation), the accuracy of the predictive models is expected to drop. This is the case for monolithic samples of 0.7 mm and 2.5 mm (Table 2), which are the extreme thicknesses of our sample data sets. For this reason, an analysis leaving these samples out was also performed, as suggested in previous studies [32], where significant differences were found for extrapolation and interpolation approaches (Table 4). As expected, more accurate estimations were found when only samples that fall within the cloud defined by the training set of data points were included in the analysis, with 100% and 47.42% of the color differences between predicted and real measured data lower than $AT_{00}$ and $PT_{00}$, respectively.

However, in the case of the layered specimens (dentine–enamel system), there are also test samples outside the cloud of data points (Table 3), (for example, dentine of 2.5 mm–enamel of 0.7 mm systems, among others) and color values predictions tend to be considerably better than extrapolations of the monolithic samples.

For this reason, the second research hypothesis was partially accepted, since, in some cases, differences in estimation accuracy were found between monolithic and layered samples, probably due to the size of the training sets and the geometry of the predictive models used—two dimensions versus three dimensions—for monolithic and layered samples, respectively.

Lastly, when comparing the performance of the predictive methods for different materials and shades (Table 4, Table 5 and Table 6), a clear difference in performance when different shades of the same material were considered was not found. However, an important difference in predictive performance was registered when different materials were considered. For both monolithic and layered samples, the estimations performed for VD and VE materials were considerably better than those obtained for VP material (Table 6). Previous studies [32] also highlighted that the performance of a predictive method for color estimation of dental materials varied between dental ceramics and DRC materials, being better for the later ones. Authors suggested that the differences in performance might come from different sample preparation procedures. However, this is not the case in this study, as all the specimens were prepared under the exact same conditions and by the same trained operator, which ensured the repeatability of the samples quality. In this sense, the type of DRC affects, to some extent, the performance of the predictive algorithms. Therefore, the third research hypothesis of this study was also partially accepted, as the performance of the proposed predictive methods is affected by the use of different types of DRC but not by the use of different shades of the same DRC.

Bearing the limitations in mind, the present study exclusively used composite materials, it is therefore necessary to include a wider range of monolithic and multi-layer materials and shades in future research. Another limitation of the present study is that only two layers (dentin composite and enamel composite) have been combined to prepare layered samples. Although using two composite layers for direct restorations has been proved to be enough to obtain a proper color match with the surrounding natural teeth [7], this might not always be valid in a clinical scenario, as dental practitioners might be confronted with the need to prepare restorations with more than two layers. The accuracy of the proposed predictive methods might be further improved (in the case of the extrapolation method) by using a different strategy when selecting the samples included in the training sets (using different sample arrangements (Figure 1) such as, diamond shape, box shape, etc.). Further studies are required in order to fully understand the behaviour of the proposed predictive models for DRC samples.

Nevertheless, the high accuracy obtained with the tested shades and materials together with the the low variability in the obtained results confirms that the proposed method for predicting the CIE-L*a*b* chromaticity coordinates of single and bi-layered samples can be successfully applied to reach an optimal color match for the studied dental materials. The predictive methods presented in this study have the potential to provide essential information of the final appearance of both monolithic and layered dental restorations and the results presented in this study could help to custom design both monolithic and layered materials with multiple industrial applications. Moreover, since it is exclusively based on CIEL*a*b* values, it is a step forward for color estimation in clinical scenarios, and therefore, leads to an improvement of chromatic accuracy of dental restorations.

## 5. Conclusions

Within the limitations of this study, it can be concluded that:

(1) Linear Regression Models can be precisely used for CIE-L*a*b* color coordinates estimation of monolithic and layered dental resin-based composites under different illuminations, that (2) there are slight differences in estimation accuracy for monolithic and layered samples and that (3) the performance of the proposed algorithm is dependent on the DRC materials but, within a group of samples manufactured using the same material, it is independent of sample shade.

## Figures and Tables

**Table 1:** Specifications of the dental resin-based composites used in this study.

Composite	Shades	Chemical Composition	Thickness (mm)
VITAPAN Excell (VE)	A2, S1, S2, S3, S4, S5	PMMA (84–86%), $SiO_{2}$ (14–15%) and Pigments (<1%)	0.7 ± 0.1; 1.0 ± 0.1; 1.5 ± 0.1; 2.0 ± 0.1 and 2.5 ± 0.1
VITAPAN Dentine (VD)	A1, A2, A3, A3.5, B2, C2, D2		
VITA Physiodens (VP)	A2		
VITA Enamel (EN)	EN1, EN2, EN3		

**Table 2:** $\Delta E_{00}$ color differences between measured and predicted CIE-L*a*b* values for all monolithic samples tested in our study.

Text Thickness	Shade						
**Dentine (mm)**	**A1**	**A2**	**A3**	**A3.5**	**B2**	**C2**	**D2**
**2.5 **	3.83	1.57	2.80	3.31	2.21	3.62	3.66
**2.0**	1.31	0.48	0.80	1.01	0.73	1.13	1.23
**1.5**	0.39	0.57	0.68	0.15	0.12	0.09	0.26
**1.0**	1.49	0.98	1.65	1.41	0.83	1.36	1.45
**0.7**	3.91	1.99	3.38	3.01	1.91	3.11	3.49

**Table 3:** $\Delta E_{00}$ color differences between measured and predicted CIE-L*a*b* values for all layered samples tested in our study.

Test Thickness	DRC Dentine (D)—Enamel (E) Systems							
**D (mm) + E (mm)**	**VE S1-EN1**	**VE S2-EN1**	**VE S3-EN2**	**VE S4-EN2**	**VE S5-EN2**	**VE A2-EN1**	**VD A2-EN3**	**VP A2-EN3**
**2.5 + 2.5**	1.09	0.80	0.55	0.51	0.54	0.89	0.67	1.58
**2.5 + 2.0**	0.65	0.77	0.44	0.48	0.59	0.90	0.49	0.89
**2.5 + 1.0**	0.26	0.19	0.31	0.30	0.27	0.42	0.25	0.61
**2.5 + 0.7**	0.67	0.30	0.36	0.33	0.31	0.20	0.12	1.06
**2.0 + 2.5**	0.42	0.10	0.10	0.14	0.25	0.26	0.46	0.17
**2.0 + 1.5**	0.22	0.31	0.35	0.29	0.32	0.38	0.13	0.41
**2.0 + 0.7**	0.36	0.17	0.41	0.27	0.29	0.35	0.42	1.16
**1.5 + 2.0**	0.26	0.56	0.15	0.11	0.21	0.49	0.27	0.60
**1.5 + 1.0**	0.15	0.12	0.60	0.29	0.28	0.22	0.36	0.75
**1.0 + 2.5**	0.20	0.54	0.30	0.30	0.13	0.43	0.15	0.87
**1.0 + 1.5**	0.75	0.09	0.16	0.16	0.33	0.19	0.18	0.58
**1.0 + 0.7**	0.35	0.27	0.54	0.40	0.34	0.19	0.59	1.86
**0.7 + 2.5**	0.38	0.73	0.34	0.35	0.11	0.40	0.14	0.26
**0.7 + 2.0**	0.60	0.29	0.21	0.40	0.18	0.79	0.26	0.69
**0.7 + 1.0**	0.14	0.33	0.27	0.62	1.17	0.51	1.02	0.50
**0.7 + 0.7**	0.89	0.77	1.10	1.21	1.36	0.99	1.71	1.74

**Table 4:** Statistics of error metrics for the extrapolation (35 specimens considered for prediction) and interpolation approach (21 specimens considered for prediction).

Method of Analysis	Variable	Mean	SD	<${\mathrm{AT}}_{00}$ (%)	<${\mathrm{PT}}_{00}$ (%)
Extrapolation (n = 35)	ΔL*	2.00	1.50		
	Δa*	0.14	0.13		
	Δb*	0.62	0.58		
	$\Delta E_{00}$	1.71	1.22	62.86	28.57
Interpolation (n = 21)	ΔL*	0.99	0.67		
	Δa*	0.09	0.07		
	Δb*	0.39	0.26		
	$\Delta E_{00}$	0.86	0.49	100	47.62

**Table 5:** Statistics of error metrics for different dental resin-based composites of A2 shade.

Layers	Variable	Mean	SD	<${\mathrm{AT}}_{00}$ (%)	<${\mathrm{PT}}_{00}$ (%)
**VE A2-EN1 **	ΔL*	0.39	0.33		
	Δa*	0.05	0.04		
	Δb*	0.34	0.25		
	$\Delta E_{00}$	0.48	0.27	100	81.25
**VD A2-EN3**	ΔL*	0.38	0.51		
	Δa*	0.06	0.04		
	Δb*	0.29	0.21		
	$\Delta E_{00}$	0.45	0.41	100	87.5
**VP A2-EN3**	ΔL*	0.78	0.60		
	Δa*	0.09	0.05		
	Δb*	0.60	0.34		
	$\Delta E_{00}$	0.86	0.51	93.75	56.25

**Table 6:** Statistics of error metrics for different shades of VITA Excell.

Layers	Variable	Mean	SD	<${\mathrm{AT}}_{00}$ (%)	<${\mathrm{PT}}_{00}$ (%)
**VE S1-EN1 **	ΔL*	0.51	0.41		
	Δa*	0.04	0.03		
	Δb*	0.24	0.20		
	$\Delta E_{00}$	0.46	0.28	100	87.50
**VE S2-EN1**	ΔL*	0.40	0.37		
	Δa*	0.03	0.02		
	Δb*	0.23	0.14		
	$\Delta E_{00}$	0.40	0.26	100	100
**VE S3-EN2**	ΔL*	0.36	0.35		
	Δa*	0.04	0.02		
	Δb*	0.23	0.15		
	$\Delta E_{00}$	0.39	0.24	100	93.75
**VE S4-EN2**	ΔL*	0.29	0.32		
	Δa*	0.05	0.03		
	Δb*	0.27	0.20		
	$\Delta E_{00}$	0.39	0.26	100	93.75
**VE S5-EN2**	ΔL*	0.20	0.16		
	Δa*	0.10	0.13		
	Δb*	0.36	0.35		
	$\Delta E_{00}$	0.42	0.35	100	87.5

## Data Availability

Not applicable.

## References

[1] Da Costa J., Fox P., Ferracane J. (2010). Comparison of various resin composite shades and layering technique with a shade guide. J. Esthet. Restor. Dent..

[2] Sadowsky S.J. (2006). An overview of treatment considerations for esthetic restorations: A review of the literature. J. Prosthet. Dent..

[3] Yadav R., Kumar M. (2019). Dental restorative composite materials: A review. J. Oral Biosci..

[4] Zhou X., Huang X., Li M., Peng X., Wang S., Zhou X., Cheng L. (2019). Development and status of resin composite as dental restorative materials. J. Appl. Polym. Sci..

[5] Reza Rezaie H., Beigi Rizi H., Rezaei Khamseh M.M., Öchsner A. (2020). Dental restorative materials. A Review on Dental Materials.

[6] Dietschi D., Ardu S., Krejci I. (2006). A new shading concept based on natural tooth color applied to direct composite restorations. Quintessence Int..

[7] Manauta J., Salat A., Putignano A., Devoto W., Paolone G., Hardan L. (2014). Stratification in anterior teeth using one dentine shade and a predefined thickness of enamel: A new concept in composite layering–Part I. Trop. Dent. J..

[8] Angerame D., Fanfoni L., De Biasi M., Bevilacqua L., Generali L. (2021). Influence of Thickness and Shade on the Color of Layered Novel Nanohybrid Composite Systems. Int. J. Periodont. Rest..

[9] dos Santos C., Rosa G.O., Quintino M.N., Alves M.F.R.P., Ribeiro S., Melo-Silva C.L. (2020). Effect of surface finishing and thickness on the translucency of zirconia dental ceramics. Ceram. Int..

[10] Shiraishi T., Watanabe I. (2016). Thickness dependence of light transmittance, translucency and opalescence of a ceria-stabilized zirconia/alumina nanocomposite for dental applications. Dent. Mater..

[11] Sampaio C.S., Gurrea J., Gurrea M., Bruguera A., Atria P.J., Janal M., Bonfante E.A., Coelho P.G., Hirata R. (2018). Dental Shade Guide Variability for Hues B, C, and D Using Cross-Polarized Photography. Int. J. Periodontics Restor. Dent..

[12] Pérez M.M., Della Bona A., Carrillo-Perez F., Dudea D., Pecho O., Herrera L. (2020). Does background color influence visual thresholds?. J. Dent..

[13] Nakhaei M., Ghanbarzadeh J., Keyvanloo S., Alavi S., Jafarzadeh H. (2013). Shade matching performance of dental students with three various lighting conditions. J. Contemp. Dent. Pract..

[14] Johnston W.M. (2009). Color measurement in dentistry. J. Dent..

[15] Dudea D., Gasparik C., Botos A., Alb F., Irimie A., Paravina R.D. (2016). Influence of background/surrounding area on accuracy of visual color matching. Clin. Oral Investig..

[16] Melgosa M., Ruiz-López J., Li C., García P.A., Della Bona A., Pérez M.M. (2020). Color inconstancy of natural teeth measured under white light-emitting diode illuminants. Dent. Mater..

[17] Carrillo-Perez F., Pecho O.E., Morales J.C., Paravina R.D., Della Bona A., Ghinea R., Pulgar R., Del Mar Pérez M., Herrera L.J. (2022). Applications of artificial intelligence in dentistry: A comprehensive review. J. Esthet. Restor. Dent..

[18] Pérez M.M., Benavides-Reyes C., Tejada-Casado M., Ruiz-López J., Lucena C. (2022). Does Backgrounds Color Influence the Appearance of Gingiva-Colored Resin-Based Composites?. Materials.

[19] Ruiz-López J., Espinar C., Lucena C., de la Cruz Cardona J., Pulgar R., Pérez M.M. (2022). Effect of thickness on color and translucency of a multi-color polymer-infiltrated ceramic-network material. J. Esthet. Restor. Dent..

[20] Pecho O.E., Ghinea R., Ionescu A.M., Cardona J.C., Della Bona A., Pérez M.M. (2015). Optical behavior of dental zirconia and dentin analyzed by Kubelka-Munk theory. Dent. Mater..

[21] Paravina R.D., Aleksić A., Tango R.N., García-Beltrán A., Johnston W.M., Ghinea R.I. (2021). Harmonization of color measurements in dentistry. Measurement.

[22] Pulgar R., Lucena C., Espinar C., Pecho O.E., Ruiz-Lopez J., Della Bona A., Perez M.M. (2019). Optical and colorimetric evaluation of a multi-color polymer-infiltrated ceramic-network material. Dent. Mater..

[23] Lucena C., Ruiz-López J., Pulgar R., Della Bona A., Pérez M.M. (2021). Optical behavior of one-shaded resin-based composites. Dent. Mater..

[24] Brandt J., Nelson S., Lauer H.C., von Hehn U., Brandt S. (2017). In vivo study for tooth colour determination—visual versus digital. Clin. Oral Investig..

[25] Chu S.J., Trushkowsky R.D., Paravina R.D. (2010). Dental color matching instruments and systems. Review of clinical and research aspects. J. Dent..

[26] Pecho O.E., Ghinea R., Alessandretti R., Pérez M.M., Della Bona A. (2016). Visual and instrumental shade matching using CIELAB and CIEDE2000 color difference formulas. Dent. Mater..

[27] Della Bona A. (2020). Color and Appearance in Dentistry.

[28] Akl M.A., Sim C.P.C., Nunn M.E., Zeng L.L., Hamza T.A., Wee A.G. (2022). Validation of two clinical color measuring instruments for use in dental research. J. Dent..

[29] Ghinea R., Pecho O., Herrera L.J., Ionescu A.M., de la Cruz Cardona J., Sanchez M.P., Paravina R.D., del Mar Perez M. (2015). Predictive algorithms for determination of reflectance data from quantity of pigments within experimental dental resin composites. Biomed. Eng. Online.

[30] Mikhail S.S., Johnston W.M. (2014). Confirmation of theoretical colour predictions for layering dental composite materials. J. Dent..

[31] Duveiller V., Gevaux L., Clerc R., Salomon J.P., Hébert M. (2020). Reflectance and transmittance of flowable dental resin composite predicted by the two-flux model: On the importance of analyzing the effective measurement geometry. Color Imaging Conf..

[32] Tejada-Casado M., Ghinea R., Perez M.M., Lübbe H., Pop-Ciutrila I.S., Ruiz-López J., Herrera L.J. (2022). Reflectance and color prediction of dental material monolithic samples with varying thickness. Dent. Mater..

[33] Tejada-Casado M., Ghinea R., Pérez M., Cardona J., Ionescu A., Lübbe H., Herrera L. (2022). Color prediction of layered dental resin composites with varying thickness. Dent. Mater..

[34] Gozalo-Diaz D., Johnston W.M., Wee A.G. (2008). Estimating the color of maxillary central incisors based on age and gender. J. Prosthet. Dent..

[35] Gómez-Polo C., Montero J., Gómez-Polo M., de Parga J.A.M.V., Celemin-Viñuela A. (2015). Natural tooth color estimation based on age and gender. J. Prosthodont..

[36] Oguro R., Nakajima M., Seki N., Sadr A., Tagami J., Sumi Y. (2016). The role of enamel thickness and refractive index on human tooth colour. J. Dent..

[37] Kose C., Oliveira D., Roulet J., Pereira P., Rocha M. (2022). Algorithm to Predict the Final Color of Leucite-Reinforced Ceramic Restorations. Dent. Mater..

[38] Wee A.G., Chen W.Y., Johnston W.M. (2005). Color formulation and reproduction of opaque dental ceramic. Dent. Mater..

[39] Mikhail S.S., Schricker S.R., Azer S.S., Brantley W.A., Johnston W.M. (2013). Optical characteristics of contemporary dental composite resin materials. J. Dent..

[40] Fairchild M.D. (2019). CIE 015:2018 Colorimetry, 4th Edition. The International Commission on Illumination, Vienna, Austria. Color Res. Appl..

[41] Westland S. (2003). Review of the CIE system of colorimetry and its use in dentistry. J. Esthet. Restor. Dent..

[42] Jost S., Ngo M., Ferrero A., Poikonen T., Pulli T., Thorseth A., Blattner P. (2017). Determination of illuminants representing typical white light emitting diodes sources. Proceedings of the CIE Midterm Meeting 2017.

[43] Maulud D., Abdulazeez A.M. (2020). A review on linear regression comprehensive in machine learning. J. Appl. Sci. Technol. Trends.

[44] Roziqin M.C., Basuki A., Harsono T. (2016). A comparison of montecarlo linear and dynamic polynomial regression in predicting dengue fever case. Proceedings of the 2016 International Conference on Knowledge Creation and Intelligent Computing (KCIC).

[45] Prasad A.K., Ahadi M., Thakur B.S., Roy S. (2015). Accurate polynomial chaos expansion for variability analysis using optimal design of experiments. Proceedings of the 2015 IEEE MTT-S International Conference on Numerical Electromagnetic and Multiphysics Modeling and Optimization (NEMO).

[46] Sharma G., Wu W., Dalal E.N. (2005). The CIEDE2000 color-difference formula: Implementation notes, supplementary test data, and mathematical observations. Color Res. Appl..

[47] (2016). Technical Report(E): Dentistry—Guidance on Color Measurements.

[48] Paravina R.D., Ghinea R., Herrera L.J., Bona A.D., Igiel C., Linninger M., Sakai M., Takahashi H., Tashkandi E., Mar Perez M. (2015). Color difference thresholds in dentistry. J. Esthet. Restor. Dent..

[49] Paravina R.D., Pérez M.M., Ghinea R. (2019). Acceptability and perceptibility thresholds in dentistry: A comprehensive review of clinical and research applications. J. Esthet. Restor. Dent..

[50] Pérez M.M., Pecho O.E., Ghinea R., Pulgar R., Della Bona A. (2019). Recent advances in color and whiteness evaluations in dentistry. Curr. Dent..

[51] Medeiros J.A., Pecho O.E., Pérez M.M., Carrillo-Pérez F., Herrera L.J., Della Bona A. (2021). Influence of background color on color perception in dentistry. J. Dent..

[52] Ruiz-Lopez J., Pulgar R., Lucena C., Pelaez-Cruz P., Cardona J.C., Perez M.M., Ghinea R. (2021). Impact of short-term dental dehydration on in-vivo dental color and whiteness. J. Dent..

[53] dos Reis Furlani Zouain Ferreira Neves Dias T., Dias N., de Campos F.U.F., Turssi C.P., do Amaral F.L.B., França F.M.G., Basting R.T. (2022). Color change after tooth bleaching with ozone and 10% ozonized carbamide peroxide for in-office use. Med. Gas Res..

[54] Pop-Ciutrila I.S., Ghinea R., Colosi H.A., Ruiz-López J., Perez M.M., Paravina R.D., Dudea D. (2021). Color compatibility between dental structures and three different types of ceramic systems. BMC Oral Health.

[55] Thornton W.A. (1999). Spectral sensitivities of the normal human visual system, color-matching functions and their principles, and how and why the two sets should coincide. Color Res. Appl..

